# Effects of Du Meridian Moxibustion Combined with Mild Moxibustion on Female Pelvic Floor Myofascial Pain Syndrome: A Retrospective Cohort Study

**DOI:** 10.1155/2022/7388864

**Published:** 2022-11-15

**Authors:** Yehong Wei, Xufeng Chen, Tianyu Wang, Xianna Dong, Zheng Zhu

**Affiliations:** ^1^Nursing Department, The Second Affiliated Hospital of Zhejiang Chinese Medical University, Hangzhou 310005, China; ^2^Department of Obstetrics and Gynecology, Hangzhou TCM Hospital, Hangzhou 310005, China; ^3^Department of Rehabilitation Medicine, The Second Affiliated Hospital of Zhejiang Chinese Medical University, Hangzhou 310005, China; ^4^TCM Nursing Clinic, The Second Affiliated Hospital of Zhejiang Chinese Medical University, Hangzhou 310005, China; ^5^Department of Urologic Surgery, The Second Affiliated Hospital of Zhejiang Chinese Medical University, Hangzhou 310005, China

## Abstract

**Objective:**

This study aimed to investigate the efficacy and safety of moxibustion in the treatment of pelvic floor myofascial pain syndrome.

**Methods:**

A total of 80 women with pelvic floor myofascial pain syndrome (cold coagulation and blood stasis type) were included in this retrospective study and divided into a moxibustion group and a drug treatment group. Patients who received Celebrex oral analgesia, health education, and lifestyle improvement were included in the drug treatment group. The patients that received Du meridian moxibustion combined with mild moxibustion, health education, and lifestyle improvement were included in the moxibustion group. The comparison of pelvic pain, the TCM clinical symptom score, and the curative effect was made between the two groups before treatment and after 1–3 weeks of treatment, respectively.

**Results:**

An intragroup comparison showed a stepwise decrease in the VAS score and the TCM clinical symptom score of the two groups during the treatment. An intergroup comparison revealed that the VAS score of the moxibustion group was lower than that of the drug treatment group, while TCM clinical symptoms and clinical efficacy significantly improved in the moxibustion group compared to those in the drug treatment group (*P* < 0.05).

**Conclusion:**

Du meridian moxibustion combined with mild moxibustion alleviates pelvic floor myofascial pain syndrome, thus helping improve women's quality of life and providing patients with a more effective and safer treatment plan.

## 1. Introduction

Chronic pelvic pain refers to a group of periodic pains caused by various organic and functional factors, with a disease course of more than 6 months. It is mainly manifested as pelvic tenderness and surrounding tissue pain, which are commonly treated with drugs or surgery [1]. Chronic pelvic pain is common in women of childbearing age, and approximately 24% of women suffer from chronic pelvic pain, accounting for 20% of the outpatient visits [2]. As a kind of chronic pelvic pain, chronic pelvic pain syndrome's (CPPS) pathogenesis is still unclear, and the course of the disease is prolonged. Thus, there are some difficulties in diagnosis and treatment of CPPS.

Pelvic floor myofascial pain syndrome, which was classified as CPPS by the European Society of Urology in 2012 [3], could be diagnosed based on a number of signs and symptoms, such as obvious tenderness points in the pelvic cavity, tension and spasm of pelvic floor myofascial, pain in the pelvic cavity and surrounding tissues, sexual intercourse pain, frequent urination, urgent urination, and other symptoms [4], and it has a serious impact on the social behavior and daily life of patients due to the prolonged course and repeated pain that may cause the dysfunction of related tissues. Currently, no clear guidelines are available for the treatment of this disease, while Western medicine usually focuses on drug treatment, and the treatment such as using NSAID is limited by obvious side effects and poor patient compliance [5]. Furthermore, the relative risk of Celebrex, a drug commonly used in clinics, was underestimated [6]. Therefore, it is urgent to find an easily implemented treatment scheme with a stable curative effect and high patient compliance for patients with pelvic floor myofascial pain syndrome. A number of treatment options including low intensity shock wave therapy [7], myofascial physiotherapy [8, 9], pelvic floor exercise [10], and other physical therapy have been shown to have certain curative effects on pelvic floor myofascial pain syndrome. However, physical therapy highly relies on the location, intensity, and frequency with obvious individual differences of patients and the lack of unified evidence-based medical evidence [11].

According to traditional Chinese medicine (TCM) theory, Yang and Qi are driving forces of human biological activities. However, pelvic floor myofascial pain syndrome shows the irregular movement of Yang and Qi[12]. The governor vessel runs in the middle of the back of the human body, which plays an important role in regulating Qi and blood of the Yang meridians in the whole body [13]. The conception vessel is of great significance to pelvic floor muscles. Moxibustion is a form of treatment derived from TCM, which is widely applied to clinical practice [14]. Burning of moxa on the governor vessel pulse was involved in moxibustion treatment. The warm effect of moxibustion on the governor vessel can promote blood circulation, remove stasis, and relieve pain [15, 16]. Moreover, several traditional Chinese medicines, such as *Asarum sieboldii*, *Rhizoma Chuanxiong*, and *Angelica sinensis pubescens* governor's moxibustion powder, show a significant effect on dispersing cold, relieving pain, and promoting blood circulation and Qi circulation, which causes an increase in the body temperature. Additionally, moxibustion instrument can provide warm stimulation for pelvic myofascial pain, and the warm effect of moxibustion is transmitted to the focus via the Ren channel, thereby facilitating the treatment of the disease. Moxibustion has the characteristics of a potential curative effect, convenience, low cost, and safety. Moxibustion has the potential to perform as a supplementary intervention for long-term health care of patients with pelvic floor myofascial pain syndrome. So far, there is no evaluation evidence of moxibustion for the treatment of pelvic floor myofascial pain syndrome. Therefore, this study conducted a retrospective study and analysis on Du meridian moxibustion and mild moxibustion for the treatment of pelvic floor myofascial pain syndrome.

## 2. Materials and Methods

### 2.1. General Information

The study included 132 patients with pelvic floor myofascial pain syndrome who received moxibustion or drug therapy in the urology or obstetrics department of a tertiary A-level-integrated traditional Chinese and Western medicine hospital between March 1, 2019, and September 30, 2020. According to the diagnostic criteria issued by the guiding principles for the clinical study of new Chinese medicine, patients diagnosed with pelvic floor myofascial pain syndrome were divided into the moxibustion group and the drug treatment group according to the treatment. When the patient seeks medical treatment, drug therapy or moxibustion therapy is chosen based on the patient's preference and the doctor's recommendation. Eighty patients with valid data after screening were eligible for this retrospective study. The patients that received Celebrex oral analgesia, health education, and lifestyle improvement were included in the drug treatment group (*n* = 40). The patients that received Du meridian moxibustion combined with mild moxibustion were included in the moxibustion group (*n* = 40).

This study was approved by the Ethics Review Committee of the Second Affiliated Hospital of Zhejiang Chinese Medical University (2022-LW-005-01) and performed in accordance with the Declaration of Helsinki (as revised in 2013).

### 2.2. Selection and Screening Criteria

Patients were diagnosed with pelvic floor myofascial pain syndrome if they met the following criteria [3]: disease differentiation in Western medicine and patients suffered from chronic pelvic pain for at least 3–6 months and had one or more pain trigger points in the pelvic floor muscle and fascia. Objective organic diseases were excluded by a laboratory test, imaging, and physical examination; (2) having a history of sexual life; and syndrome differentiation of traditional Chinese medicine. Pelvic floor myofascial pain syndrome is characterized by the main symptoms, including hidden pains in the perineum, suprapubic bone, and lower abdomen, based on the description of the main and secondary symptoms of CPPS in the guiding principles for the clinical study of new Chinese medicine [12]. Secondary symptoms of pelvic floor myofascial pain syndrome include (a) urinary tract irritation, (b) loose stool, (c) enlarged cold pain in the perineum and lower abdomen, and (d) traditional Chinese medicine, which is divided into two types: cold coagulation and blood stasis, accompanied by the cool foot, heart, and tongue. The tongue coating is white and dark, there are ecchymosis spots on it, and the pulse string is heavy. The diagnosis was made if patients had the main symptom as well as two or more secondary symptoms.

Inclusion criteria were as follows: (1) aged 18–56 years; (2) cold coagulation and blood stasis type based on TCM syndrome differentiation and classification, with clear consciousness and availability for clinical data collection and scoring with the scale. No disease-related medications were given within 1 month.

Exclusion criteria were as follows: (1) those complicated with diseases of the lumbar spine, digestive system, kidneys, or urinary tract stones; (2) those with open wounds, skin infections, ulcers, and so forth; (3) pregnant or lactating women; (4) those with severe primary diseases and mental diseases; (5) those who were allergic to this treatment; (6) deterioration of condition after governor vessel treatment; (7) patients with poor compliance and those who were unable to complete treatment or required other treatment; and (8) patients who displayed serious adverse reactions, other accident treatments, or other treatments were needed.

### 2.3. Experimental Method

The patients were divided into two groups in this study, i.e., the drug treatment group and moxibustion group. Forty patients in the drug treatment group received the following basic treatment: Celebrex (Pfizer) 100 mg twice a day was administered as the treatment during health education. Wind and cold were avoided, and the pelvic cavity was kept warm. A light diet was recommended. River crab, balsam pear, millet, mung bean, and other cool-natured foods were avoided.

The moxibustion group underwent Du meridian moxibustion combined with mild moxibustion, and the other treatment method was the same as that in the drug treatment group, except that no medication was taken.

### 2.4. Moxibustion Treatment on the Governor Vessel

**Figure 1:**
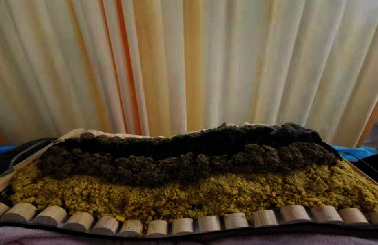
When the patient is in the prone position, the governor's moxibustion powder is spread evenly on the Dazhui points to Balu points of the governor's meridian in a linear shape, and mulberry paper is used to cover the governor's moxibustion powder. Ginger wool has a length, width, and height of at least 3.5 cm × 3.0 cm × 2.5 cm. Ginger wool is placed in the center of the mulberry paper in a trapezoid shape, which has a length, width, and height of at least 3.5 cm × 3.0 cm × 2.5 cm. A triangular, pyramid-shaped moxa cone of a suitable size is placed on ginger wool.

**Figure 2:**
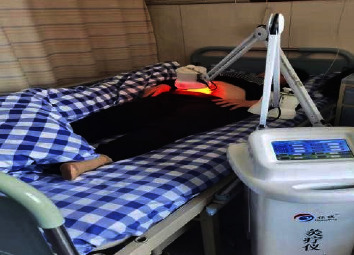
Mild moxibustion with a moxibustion instrument was performed on the patient at the pelvic tender point in a lying position.

As depicted in Figure 1, moxibustion treatment on the Governor Vessel is a characteristic external treatment method of traditional Chinese medicine, in which powder moxibustion, ginger lining, and moxa velvet moxibustion were used to make foam on the spine of the governor vessel [17, 18]. As shown in Figure 2, the moxibustion instrument is manufactured according to the principle of traditional moxibustion and adopts electronic, control, and sensing technologies. Compared with the traditional moxibustion treatment, the one that uses this smokeless moxibustion instrument (JLY-II, Huaicheng, Taiyuan, China) without open fire can significantly reduce the discomfort of patients. Moreover, the moxibustion apparatus is equipped with an infrared appliance to relieve pain.

Patients received moxibustion treatment once every three day (every interval of 2 days) in the TCM nursing clinic. Before the treatment, a certain proportion of the Du moxibustion powder (Qinjiao: Xixin: Fangfeng: Chuanxiong: Duhuo = 2 : 2: 2 : 3), mulberry paper, ginger mud, and moxa column spare were prepared. Each patient underwent moxibustion in the prone position on the moxibustion bed with a bare back, and moxibustion sites were cleaned and disinfected before operation. The Du moxibustion powder was evenly spread in a line shape from the point GV14 of the Du meridian to eight influential points. We took the mulberry paper cover off the Du moxibustion powder and made ginger mud with a 3.5 cm × 3.0 cm × 2.5 cm trapezoidal paving in the center of mulberry paper. We put the appropriate size of the triangular, pyramid-shaped moxa cone on ginger mud and an ignited moxa cone and let it undergo spontaneous combustion, and continuous moxibustion was implemented 3 times.

Mild moxibustion was performed daily at the pelvic tenderness point by using a moxibustion instrument equipped with infrared radiation. The probe of the moxibustion instrument was placed above the tenderness point approximately 15 cm away, and power was switched on. The temperature was set at 240°C, and the intensity was 2. A 30-minute treatment was performed with the probe of the moxibustion instrument 10 cm away from the body surface, and the treatment temperature on the body surface was approximately 50°C. The treatment was provided in a comfortable and warm environment, and privacy was protected during the treatment.

### 2.5. Outcome Measurements

The two groups of patients were investigated in the form of questionnaires, and the investigation was carried out by the trained attending physician and TCM nursing outpatient nurses before treatment and after 1–3 weeks of treatment. Evaluation was performed by using the following three methods.

#### 2.5.1. VAS Scoring

Patients in the two groups were scored with a visual analog scale/score (VAS) before treatment and after 1–3 weeks of treatment, respectively [18]. VAS scoring was used to evaluate the pain degree of patients. A 10 cm horizontal line was drawn on the paper. The left-most side of the horizontal line indicates painlessness (score of 0), and the right-most side indicates severe pain (score of 10). The middle part of the line corresponds to varying degrees of pain. Patients were asked to mark the transverse line according to their self-feeling, and the degree of pain was classified as follows: 0–2, comfort; 3-4, slight discomfort; 5-6, moderate discomfort; 7-8, severe discomfort; and 9-10, extreme discomfort.

#### 2.5.2. TCM Clinical Symptom Scoring

TCM clinical symptom scoring was performed according to the expression of TCM clinical symptoms of the disease in the guiding principles for clinical research of new traditional Chinese medicine. The scores were assessed based on abdominal perineum and lumbar sacral pain, aggravation of pain in cold weather, fear of cold in the lower perineum, frequent micturition or poor micturition, and urethral pricking pain with urination. TCM clinical symptom scores were categorized into four groups: 0, none; 2, occasional; 4, sometimes; and 6, often.

#### 2.5.3. Evaluation of the Efficacy Index

To evaluate the curative effect, the curative effect index was calculated after 3 weeks of treatment as follows [19]: curative effect index = ((TCM clinical symptom score before treatment—TCM clinical symptom score after treatment)/TCM clinical symptom score before treatment) × 100%. The curative effect index was used to define the curative effect as follows: no less than 95%, clinically cured; 70%–95%, markedly effective; 30%–70%, effective; and less than 30%, ineffective.

All of the above information and data were extracted during the patient's treatment period. Assessors did not intentionally guide the patient's preference during the evaluation process.

### 2.6. Statistical Analysis

Data were statistically processed by using SPSS 22.0 software. The homogeneity of data between the two groups was tested. The measurement data and counting data were analyzed using the sample *t*-test and *χ*^2^ test, respectively. The generalized linear model (GLM) for the repeated measure ANOVA test was used to determine the longitudinal variation in total samples. Group comparisons between the VAS score and TCM symptom score at each interval were made by the Mann–Whitney *U* test. Statistical significance was considered at *P* < 0.05.

## 3. Results

### 3.1. Participants and Baseline Characteristics

Data from 80 patients were included in the analysis. As shown in Table 1, there were no statistically significant differences in baseline data, such as age, the degree of education, and the course of disease, between the two groups.

### 3.2. Primary Outcomes

**Figure 3:**
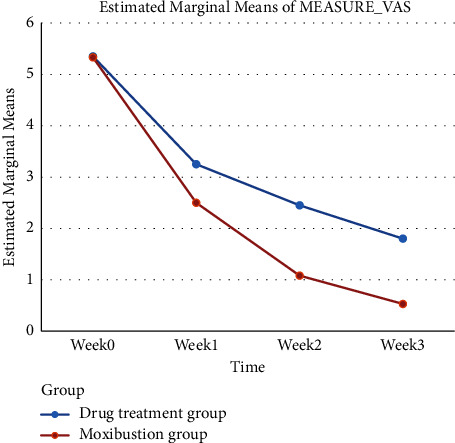
The line chart of VAS scores of patients with different treatment methods over time. VAS, visual analog scale/score.

As summarized in Table 2, Figure 3, and Figure S1, VAS scores of pelvic floor myofascial pain syndrome were correlated with treatment and time (*P* < 0.05). Moreover, there were significant differences in the scores between the two groups at each time point after treatment, indicating that the main effect of treatment measures and time on VAS scores of pelvic floor myofascial pain syndrome is remarkable (*P* < 0.05).

### 3.3. Secondary Outcomes

**Figure 4:**
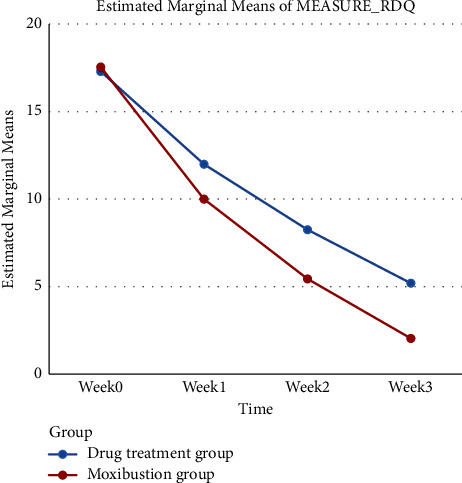
The line chart of TCM scores of patients with different treatment methods over time.

As shown in Table 3 and Figure 4, the TCM syndrome score of pelvic floor myofascial pain syndrome was correlated with treatment and time (*P* < 0.05). Moreover, there were significant differences in TCM symptom scores between the two groups at each time point after treatment, indicating that the main effect of treatment measures and time on TCM symptom scores is pronounced (*P* < 0.05).

### 3.4. Exploratory Outcomes

As presented in Table 4, although both the drug treatment and moxibustion groups had favorable curative effects, the curative effect of the moxibustion group was significantly better than that of the drug treatment group (*P* < 0.05).

### 3.5. Adverse Reaction

Adverse reactions included skin allergy, scald, pain, and other discomforts. No serious adverse events occurred during the intervention period in both groups.

## 4. Discussion

This study was based on the study of the effects of performing Du meridian moxibustion with mild moxibustion on urinary, bowel, and sexual function in women with pelvic floor myofascial pain syndrome, and when data were compiled, it was found that Du meridian moxibustion with mild moxibustion positively reduced the patients' pain and TCM clinical symptoms. In this study, we showed that a more significant decrease in VAS pain scores or TCM symptom scores during the treatment was found in the moxibustion group than that in the drug treatment group, while the curative effect in the moxibustion group was significantly better than that in the drug treatment group. These observations indicated that compared with oral administration of Celebrex and therapeutic lifestyle change, moxibustion treatment can be more conducive to alleviating uncomfortable symptoms of patients with pelvic floor myofascial pain syndrome. Meanwhile, no adverse reactions occurred during 3 weeks of treatment, and safety can be guaranteed. This finding is consistent with those of the previous reports [20].

As a common gynecological disease, CPPS has a serious impact on the social behavior and daily life of patients due to the prolonged disease course and repeated pain. Pelvic floor myofascial pain affects both women and men and has been found to be caused by potential weakness, overuse, trauma (i.e., vaginal delivery, especially perineal laceration or obstetric anal sphincter injury) or compensatory injury by complex and diverse mechanisms, characterized by pressure points [21, 22]. The myofascial tenderness point can not only limit the activity of local tissues but also cause pain at the primary site and distant area. In order to relieve pelvic pain, taking drugs is often the first choice. Celebrex, commonly used in our department to treat CPPS, is a nonsteroidal anti-inflammatory drug with analgesic and anti-inflammatory effects, and it can inhibit prostaglandin production by inhibiting cyclooxygenase-2 [23] and relieve pelvic pain, which can avoid the severe gastrointestinal side effects of traditional NSAIDs. In the present study, three weeks of treatment with Celebrex led to a decrease in VAS scores of patients in both groups as well as in TCM symptom scores. The reduction in the VAS was more pronounced in the drug treatment group of this study than that in previous studies on male patients [24], and the reason may be attributed to the good sensitivity of the patients to the drug assessed from the time of their first dose of Celebrex. However, the long-term use of Celebrex is not a good choice. Notably, we observed more significant pain relief in the moxibustion group than in the drug treatment group according to the VAS scores. On the basis of the theory of traditional Chinese medicine, moxibustion treatment on the governor pulse can regulate the cold state of patients as a whole, for the governor pulse starts from the pelvic cavity and commands the Yang Qi of the whole body. For the CPPS (the type of cold coagulation and blood stasis) included in this study, it takes meridians as carriers to stimulate human Yang, warm the pelvic cavity, and activate blood circulation, thereby achieving the effect of removing cold, dampness, and pain [25]. As expected, a more significant decrease in the TCM symptom scores was identified in the moxibustion group than that in the drug treatment group.

Studies have shown [26, 27] that the onset of pelvic myofascial pain syndrome results from excessive activity of local pelvic muscles, which causes tissue hypoxia and ischemia as well as the accumulation of lactic acids, thereby forming a myofascial pain trigger point. It has been shown that the myofascial trigger point is the cause of many nonorganic pain syndromes of neuromuscular fibers [28]. Myofascial trigger points are similar to Ashi acupoints, which “consider pain to be acupoints” in traditional Chinese medicine acupuncture and moxibustion. Of the 255 trigger points proposed by Simons and Tavell, 194 (76%) have the same pain involved route as the meridian direction of the corresponding acupoint [29]. Modern research studies have demonstrated that local stimulation of myofascial trigger points exerts positive effects on improving local blood circulation and eliminating pain and other symptoms [30].

This study presents a number of strengths. First, in the moxibustion group, the moxibustion instrument was employed to perform mild moxibustion at the pelvic myofascial trigger point, the corresponding acupoints or Ashi points in the pelvic cavity, which is a convenient treatment that can be carried out without professional doctors. Its warmth and heat effect can effectively alleviate local cold and coagulation stagnation, reduce tissue hypoxia caused by excessive activity of local muscles in the pelvic cavity, and relax the surrounding muscles, thereby accelerating local metabolism, promoting the excretion of metabolites and pain-causing factors, and relieving local pain. Second, the moxibustion instrument has infrared treatment function. Although this study did not explore the role of infrared in the treatment of pelvic floor myofascial pain, a number of studies have shown [8, 31, 32] that infrared radiation is conducive to reducing pain. Consistently, we found that moxibustion at the trigger points of the governor vessel meridian and pelvic myofascial was more effective than Celebrex treatment and lifestyle change in patients with pelvic floor myofascial pain syndrome (Table 4). Besides, this study is the first to treat CPPS patients with the cold coagulation and blood stasis type using moxibustion. Patients with the cold coagulation and blood stasis type were screened through TCM diagnosis in the system of TCM, and TCM symptom scores were used in diagnosis, combined with VAS scores, which made the study more targeted.

Limitations exist in our study. First, as a single-center, nonrandomized, and retrospective study, it may be hindered by selection bias. Second, detection bias may exist due to the subjective measurement. Third, this study lacked a longer follow-up of patient recovery.

## 5. Conclusion

Governor vessel moxibustion combined with mild moxibustion can improve the body as a whole and effectively treat pelvic floor myofascial pain syndrome of the cold coagulation and blood stasis type, while achieving the positive curative effect. This pelvic floor myofascial pain syndrome therapeutic strategy provides a theoretical and practical basis for the external treatment of pelvic floor myofascial pain syndrome of the cold coagulation and blood stasis type by traditional Chinese medicine moxibustion, thus possessing a broad clinical application prospect. However, based on the above limitations, we need a well-conducted randomized controlled trial to further evaluate its efficacy.

## Figures and Tables

**Table 1:** Comparison of baseline data between the two groups.

Group	Number of cases	Educational level (cases)		Age (years)	Course of the disease (month)
		Bachelor degree or above	Educational background of senior middle school or below		
Drug treatment group	40	9	31	46.43 ± 7.355	12.28 ± 4.63
Moxibustion group	40	7	33	44.68 ± 9.030	12.83 ± 5.58
*T* (c2) value		0.313^*a*^		0.950	−0.479
*P* value		0.576		0.345	0.633

Note: compared with the drug treatment group at the same time point, ^*a*^*P* < 0.01. Patients with high educational level received university education or above, and others were at low educational level.

**Table 2:** Pain scores of the VAS ($\bar{X}\pm s$).

Group	Number of cases	VAS			
		Week 0	Week 1	Week 2	Week 3
Drug treatment group	40	5.35 ± 1.001	3.25 ± 0.707^*b*^	2.45 ± 0.504^*b*^	1.80 ± 0.464^*b*^
Moxibustion group	40	5.33 ± 0.944^*a*^	2.50 ± 0.555^*ab*^	1.08 ± 0.616^*ab*^	0.53 ± 0.506^*ab*^

*F*	*F* (time by group) = 21.499, *F* (between groups) = 70.148, *F* (time) = 765.045				
*P*	*P* (interactive) = 0.001, *P* (between groups) = 0.001, *P* (time) = 0.001				

Note: ^*a*^*P* < 0.01, compared with the drug treatment group at the same time point; ^*b*^*P* < 0.01, compared with before treatment.

**Table 3:** TCM symptom score ($\bar{X}\pm s$).

Group	Number of cases	TCM symptom score			
		Week 0	Week 1	Week 2	Week 3
Drug treatment group	40	17.30 ± 3.911	12.00 ± 2.970^*b*^	8.25 ± 2.529^*b*^	5.20 ± 2.066^*b*^
Moxibustion group	40	17.55 ± 3.775^*a*^	10.00 ± 3.389^*ab*^	5.45 ± 2.353^*ab*^	2.05 ± 1.535^*ab*^
*F*	*F* (time by group) = 17.889, *F* (between groups) = 11.239, *F* (time) = 1087.172				
*P*	*P* (interactive) = 0.001, *P* (between groups) = 0.001, *P* (time) = 0.001				

Notes: ^*a*^*P* < 0.01, compared with the drug treatment group at the same time point; ^*b*^*P* < 0.01, compared with before treatment. ^*b*^*P* < 0.01

**Table 4:** The clinical efficacy between the two groups (case (%)).

	Number of cases	Cure	Remarkable effect	Effective	Invalid
Drug treatment group	40	6	18	10	6
Moxibustion group	40	12	20	6	2
*U*	584.000				
*P*	0.026				

## Data Availability

The data used to support the findings of this study are available from the corresponding author upon request.

## Supplementary Materials

Supplementary Materials
*Figure S1.* Reduction in VAS scores of patients with different treatment methods over time. VAS, visual analog scale/score.
