# Supplementary material for: Effects of Du Meridian Moxibustion Combined with Mild Moxibustion on Female Pelvic Floor Myofascial Pain Syndrome: A Retrospective Cohort Study
Source: Evid Based Complement Alternat Med. 2022 Nov 15;2022:7388864. doi: 10.1155/2022/7388864 (PMC9681563; doi:10.1155/2022/7388864)
Supplement: Supplementary file 1 [file 7388864.f1.docx]

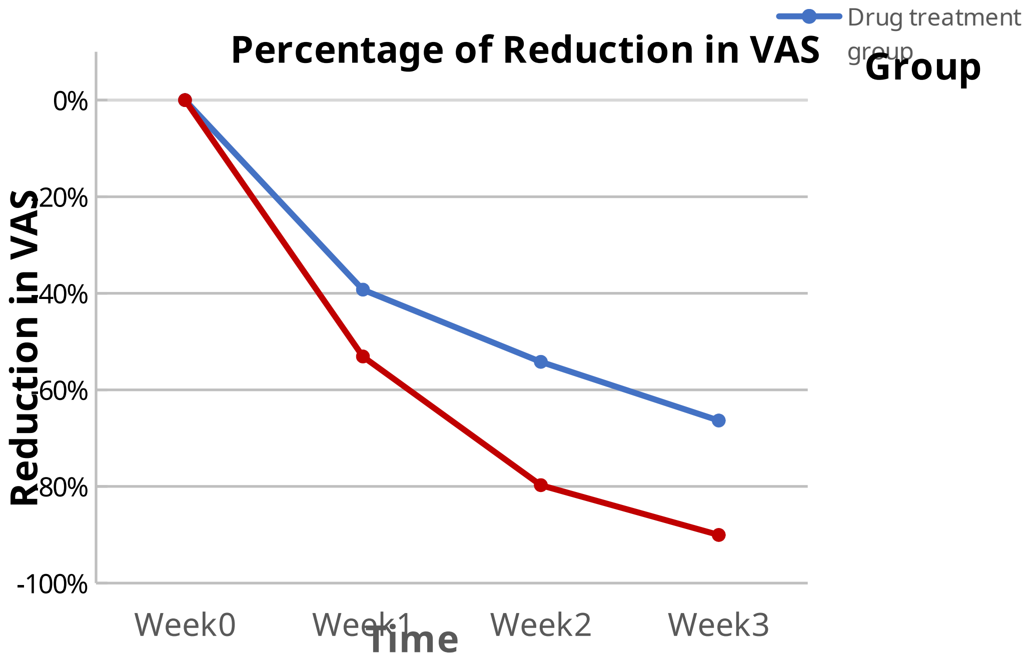
Percentage of Reduction in VAS
Drug treatment group
Group
0%
-20%
-40%
-60%
-80%
-100%
Reduction in VAS
Week0
Week1
Week2
Week3
Time
